# Psychological barriers to bystander AED use: Associations of empathy with willingness to perform bystander rescue behaviors

**DOI:** 10.1016/j.resplu.2026.101394

**Published:** 2026-06-23

**Authors:** Koichi Takaishi, Hideko Kono, Masaya Onuma, Michi Fukushima, Ryosuke Takeuchi

**Affiliations:** a Graduate School of International Social Sciences, Yokohama National University, 79-4 Tokiwadai, Hodogaya-ku, Yokohama-shi 240-8501, Japan; b Graduate School of Economics and Management, Tohoku University, 27-1 Kawauchi, Aoba-ku, Sendai-shi 980-8576, Japan

**Keywords:** Empathy, AED, OHCA, Bystander

## Abstract

**Background:**

Despite public access to automated external defibrillators (AEDs), bystander use remains limited. The psychological factors influencing rescue behaviors are not well understood.

**Objective:**

To examine how three empathy dimensions (empathic concern, perspective-taking, and personal distress) are associated with willingness to intervene in bystander rescue behaviors.

**Methods:**

A cross-sectional survey of laypersons from Japan recruited using a nationwide online research panel operated by a commercial company was conducted using hypothetical train-station cardiac arrest scenarios. Participants rated their willingness to perform three rescue behaviors—running up to the victim or calling for help, retrieving an AED, and using an AED—on a 5-point Likert scale, with high willingness indicated by scores of 4–5. Univariable and multivariable logistic regression analyses were performed to examine associations between empathy dimensions and willingness to intervene.

**Results:**

Overall, 1050 participants were included. High willingness was most frequent for running up to the victim or calling for help (72.6%), followed by retrieving an AED (40.6%), and using an AED (25.7%). Higher empathic concern was associated with greater willingness across all three rescue behaviors. Similar patterns were observed in univariable analyses and after multivariable adjustment. Perspective-taking was positively associated mainly with AED-related behaviors, whereas personal distress was inversely associated with willingness to retrieve and use an AED.

**Conclusions:**

Distinct empathy dimensions may play different roles depending on the type of rescue behavior. Empathic concern and perspective-taking may facilitate AED-related actions, whereas personal distress may represent a psychological barrier. Interventions targeting emotion regulation and perspective-taking may help improve bystander AED use.

## Highlights

• Participants had the highest willingness for running up to the victim or calling for help, and the lowest for AED use.
• Empathy dimensions were associated with willingness during a simulated cardiac arrest scenarios.
• Among the empathy dimensions, empathic concern was positively associated with willingness across all rescue behaviors.
• Perspective-taking was positively associated with willingness to retrieve and use an AED.
• Personal distress was independently associated with lower willingness for AED-related actions.

## 1 Introduction

Survival after out-of-hospital cardiac arrest (OHCA), a major cause of sudden death worldwide, depends on early defibrillation when a shockable rhythm is present. International resuscitation guidelines consistently emphasize bystander cardiopulmonary resuscitation (CPR) and prompt use of an automated external defibrillator (AED) as core components of the chain of survival.^1^, ^2^, ^3^ Evidence from public access defibrillation (PAD) programs shows that rapid bystander defibrillation substantially improves survival and neurological outcomes.^4^, ^5^ Japan is recognized as a global leader in AED dissemination and PAD system development. Nationwide registry-based studies indicate that early bystander AED use is strongly associated with improved outcomes following OHCA.^5^, ^6^, ^7^ Nevertheless, a substantial gap persists between availability and use during emergencies.^5^, ^6^, ^7^, ^8^ According to Japan’s Fire and Disaster Management Agency, 28,354 bystander-witnessed cases of cardiogenic OHCAs were reported nationwide in 2023. Bystander CPR was provided in 16,927 cases (59.7%), whereas bystander defibrillation using an AED occurred in only 1407 (5%).^8^ Importantly, outcomes were markedly better with bystander defibrillation, with a 1-month survival rate of 54.2% and a favorable neurological outcome rate of 44.9%.^8^ Thus, despite widespread AED availability and its demonstrated effectiveness, initiating AED use in real-world emergencies remains a challenge for bystanders in Japan.

Recent guidelines and consensus statements from the American Heart Association and the European Resuscitation Council increasingly emphasize the central role of lay bystanders in improving OHCA survival.^1^, ^2^, ^3^ Accordingly, although prior research focusing on determinants of bystander intervention, including CPR and AED training, AED locations knowledge, legal awareness, and self-efficacy,^9^, ^10^, ^11^, ^12^ showed that training and higher perceived competence were associated with a greater willingness to intervene, these factors alone may be insufficient to close the gap between AED availability and actual use.

Japanese experts have recently emphasized the need to reconsider conventional approaches to basic life support (BLS) education. In a national expert review; Japanese researchers noted that traditional BLS training, primarily focusing on technical skills (chest compressions and AED use), may be insufficient to consistently prompt intervention in real emergencies.^13^ Incorporating psychological factors such as the bystander effect into BLS training programs is also essential. This expert perspective provides important contextual support for examining the psychological mechanisms underlying bystander behavior, particularly in Japan, where structural preparedness is high but behavioral uptake remains limited.

One limitation of existing research is that bystander intervention has often been examined as a single binary decision, despite evidence suggesting that different forms of helping behavior may involve qualitatively distinct psychological demands. Social psychological research has long conceptualized helping in emergencies as a process in which potential helpers must overcome multiple psychological barriers (interpreting the situation, assuming responsibility, and managing perceived costs and uncertainty). From an action-threshold perspective, different rescue behaviors may require different levels of motivation, confidence, and emotional regulation.^14^, ^15^

In real-world cardiac arrest situations, bystanders may engage in a range of actions that vary in required effort, responsibility, and emotional burden: approaching the victim, retrieving an AED, or operating the device. Each action may impose qualitatively different psychological burdens and be influenced by distinct facilitators and inhibitors. Recent studies have identified a bystander effect on AED-related rescue intentions, highlighting the importance of psychological and social factors in decisions involving AED use.^16^

This perspective suggests that psychological factors may differentially influence willingness across rescue actions with varying behavioral demands. Empathy, in particular, may play distinct roles depending on the responsibility and emotional engagement required. Empathy, a potentially important yet underexplored psychological construct in this context, is a multidimensional construct involving the capacity to understand and/or share another person’s emotional state, commonly conceptualized as comprising both affective and cognitive components.^17^, ^18^, ^19^ In this framework, empathic concern reflects other-oriented feelings of compassion or concern for a person in distress, perspective-taking refers to the cognitive tendency to adopt another person’s viewpoint, and personal distress reflects a self-oriented aversive emotional response to another’s suffering.^18^, ^19^, ^20^ However, few studies have examined how specific dimensions of empathy relate to willingness to engage in different forms of bystander intervention during OHCA. Other-oriented dimensions such as empathic concern and cognitive perspective-taking facilitate prosocial and helping behaviors, whereas self-oriented personal distress may induce avoidance and inhibit action.^18^, ^19^, ^20^ These distinctions may be especially relevant in emergencies, when emotional arousal may intensify as intervention demands increase.

Herein, rescue behavior was operationalized as three distinct actions that differ in required involvement and perceived responsibility: running up to the victim or calling for help, retrieving an AED, and using an AED. We then examined how empathic concern, perspective-taking, and personal distress were associated with willingness to perform each action. We hypothesized that empathic concern and perspective-taking would facilitate willingness, whereas personal distress would inhibit it, particularly for AED-related actions.

## 2 Methods

### 2.1 Study design and participants

A nationwide, online, cross-sectional survey was conducted to assess the willingness of laypersons to intervene when witnessing a possible cardiac arrest while alone at a train station. Participants were randomly invited from a commercial survey panel using Questant (MACROMILL, INC., Tokyo, Japan), which uses a general-purpose online research panel of registered individuals who have agreed to participate in a broad range of surveys not limited to health-related topics. Registered residents from all 47 Japanese prefectures were randomly sampled, with recruitment designed to broadly represent both sexes and different age groups. To specifically examine rescue behavior among laypersons, healthcare professionals (including physicians, nurses, and paramedics) and station staff (including station security guards) were excluded, although some prior studies have considered them bystanders when acting outside their professional roles. The study was conducted in accordance with the Declaration of Helsinki. Participants were provided with a detailed information sheet describing the study objectives and procedures. Participation was voluntary, responses were kept anonymous, and informed consent was obtained prior to survey completion.

Invitation e-mails containing a link to the online questionnaire were sent to 1480 registered individuals on August 14, 2023. Data collection was planned to continue until approximately 1000 valid responses were obtained. A total of 1123 responses were received (response rate: 75.8%). After excluding 38 incomplete responses and 35 respondents who were identified as physicians or station staff, the final sample consisted of 1050 participants. Ethics approval was obtained from the Yokohama National University Human Research Ethics Committee (#2023–13).

### 2.2 Survey instrument and rescue behavior scenarios

A structured questionnaire incorporating a hypothetical scenario was developed to assess bystanders’ willingness to intervene when witnessing a cardiac arrest at a train station. Prior to responding, participants were provided with brief information about possible cardiac arrest and AED effectiveness. They were then presented with the following vignette:

“Imagine that you were alone on a station platform when a stranger collapsed in front of you. The person was unresponsive and his breathing did not seem to be normal. How do you think you would act?”

Rescue behavior was assessed as three distinct intervention actions with differing psychological demands:

(1) Rescue by running up to the victim or calling for help,
(2) Rescue by retrieving an AED,
(3) Rescue by using an AED.

For each rescue behavior, participants rated their willingness on a 5-point Likert scale ranging from 1 (unwilling) to 5 (willing).

### 2.3 Measures of empathy

In this study, three dimensions of empathy—empathic concern, perspective-taking, and personal distress—were assessed using three subscales from the Interpersonal Reactivity Index (IRI).^21^ A validated Japanese version of the IRI developed by Himichi et al.^22^ was used. Although the original IRI includes four subscales, we focused on these three dimensions because they were considered most relevant to actual bystander intervention during cardiac arrest. Participants responded to all items on a 5-point Likert scale. Empathic concern was assessed using 4 items (Cronbach’s *α* = 0.74), perspective taking using 5 items (Cronbach’s *α* = 0.75), and personal distress using 5 items (Cronbach’s *α* = 0.85). For descriptive analyses and visualization, each empathy dimension was categorized into three levels: low (scores 1–2); neutral (score 3); and high (scores 4–5). For regression analyses, all empathy dimensions were treated as continuous variables. To enhance transparency, the specific items used for each subscale are provided in Supplementary Table S1.

### 2.4 Definition of the three outcome variables

For the main analysis, each of the three rescue behaviors was treated as a separate outcome variable. For each behavior, willingness was dichotomized as follows:

• High willingness: Likert scores of 4–5
• Not high willingness: Likert scores of 1–3

This dichotomization was applied to identify participants reporting a clearly affirmative willingness to intervene, as in prior research using similar threshold-based classifications of willingness in emergency helping contexts. No composite or overall willingness score was calculated.

### 2.5 Statistical analysis

Descriptive statistics were used to summarize participant characteristics and the proportions reporting high willingness for each rescue behavior. Logistic regression analyses were conducted to estimate adjusted odds ratios (aORs) and 95% confidence intervals for high willingness to perform each rescue behavior. All models simultaneously included age, sex, AED training experience, empathic concern, perspective-taking, and personal distress. To assess potential multicollinearity among the explanatory variables, variance inflation factors (VIFs) were examined. All VIF values were below the commonly accepted threshold, suggesting multicollinearity was not relevant herein.

Descriptive statistics were calculated using standard spreadsheet software. Binary logistic regression analyses were performed using SPSS (version 27) software. As a sensitivity analysis, ordinal logistic regression models were fitted using the original 5-point willingness scale.

## 3 Results

### 3.1 Participant characteristics

A total of 1050 respondents were included in the analysis. The modal age group fell within the 50–59-year, and 63.6% were male. Overall, 60.5% of participants reported having attended AED training. The participant characteristics are summarized in Table 1.

**Table 1:** Participant characteristics (*N* = 1050).

**Characteristic**	**Category**	***n***	**(%)**
Sex	Female	382	(36.4)
	Male	668	(63.6)

Age group (years)	Under 29	88	(8.4)
	30–39	147	(14.0)
	40–49	229	(21.8)
	50–59	259	(24.7)
	60 and over	219	(20.9)
	Missing	108	(10.3)

AED training experience	No	415	(39.5)
	Yes	635	(60.5)

Values are shown as *n* (%).

*Abbreviation:* AED, automated external defibrillator.

### 3.2 Willingness across rescue behaviors

The proportion of respondents reporting high willingness was lower for behaviors requiring greater involvement (Table 2). High willingness was highest for running up to the victim or calling for help (72.6%), followed by retrieving an AED (40.6%) and using it (25.7%).

**Table 2:** Proportion of respondents reporting high willingness (Likert scale score 4–5).

**Rescue behavior**	**High willingness, *n* (%)**
Rescue by running up to the victim or calling for help	762 (72.6)
Rescue by retrieving an AED	426 (40.6)
Rescue by using an AED	270 (25.7)

*Abbreviation:* AED, automated external defibrillator.

### 3.3 Associations between empathy and willingness

**Fig. 1:**
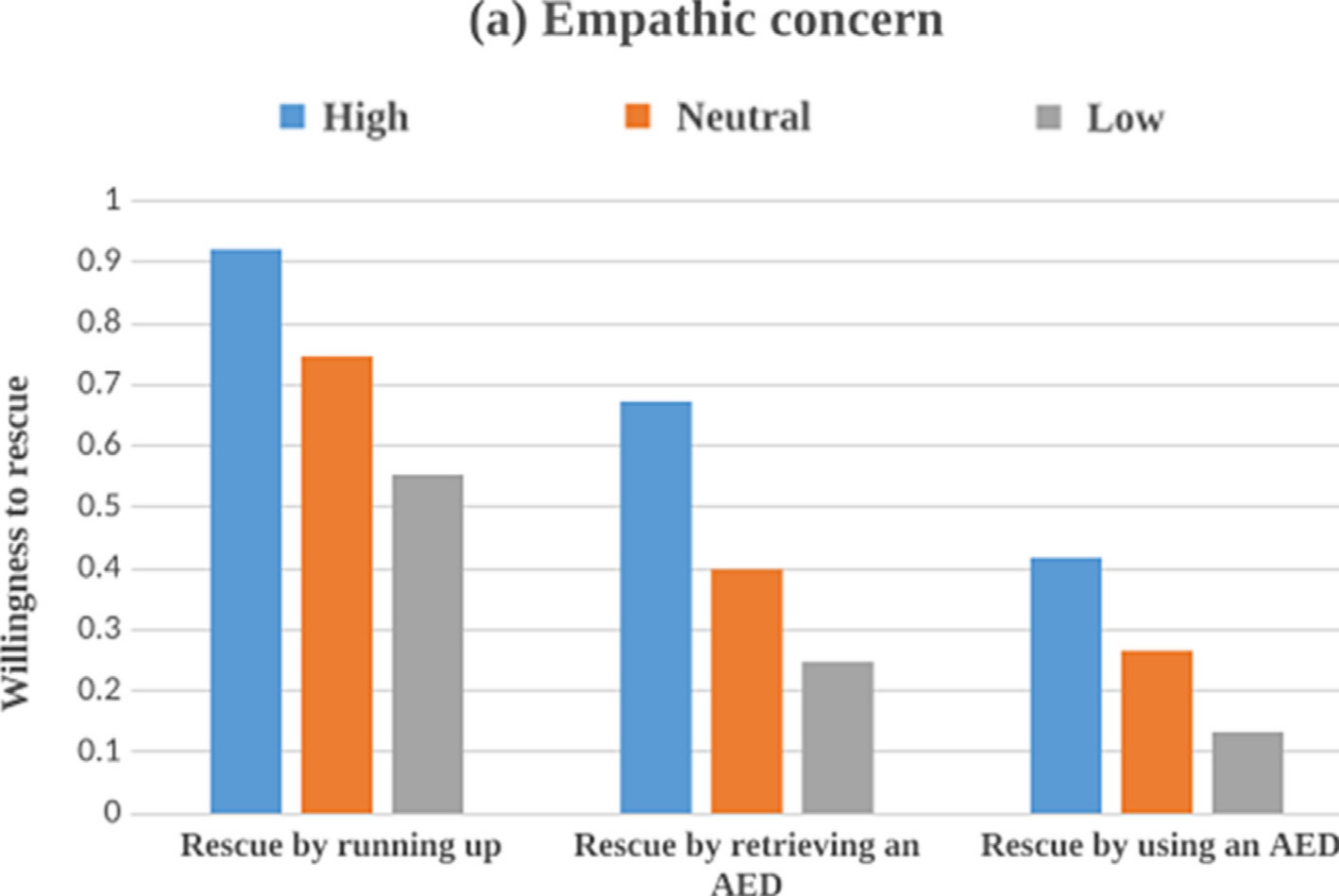

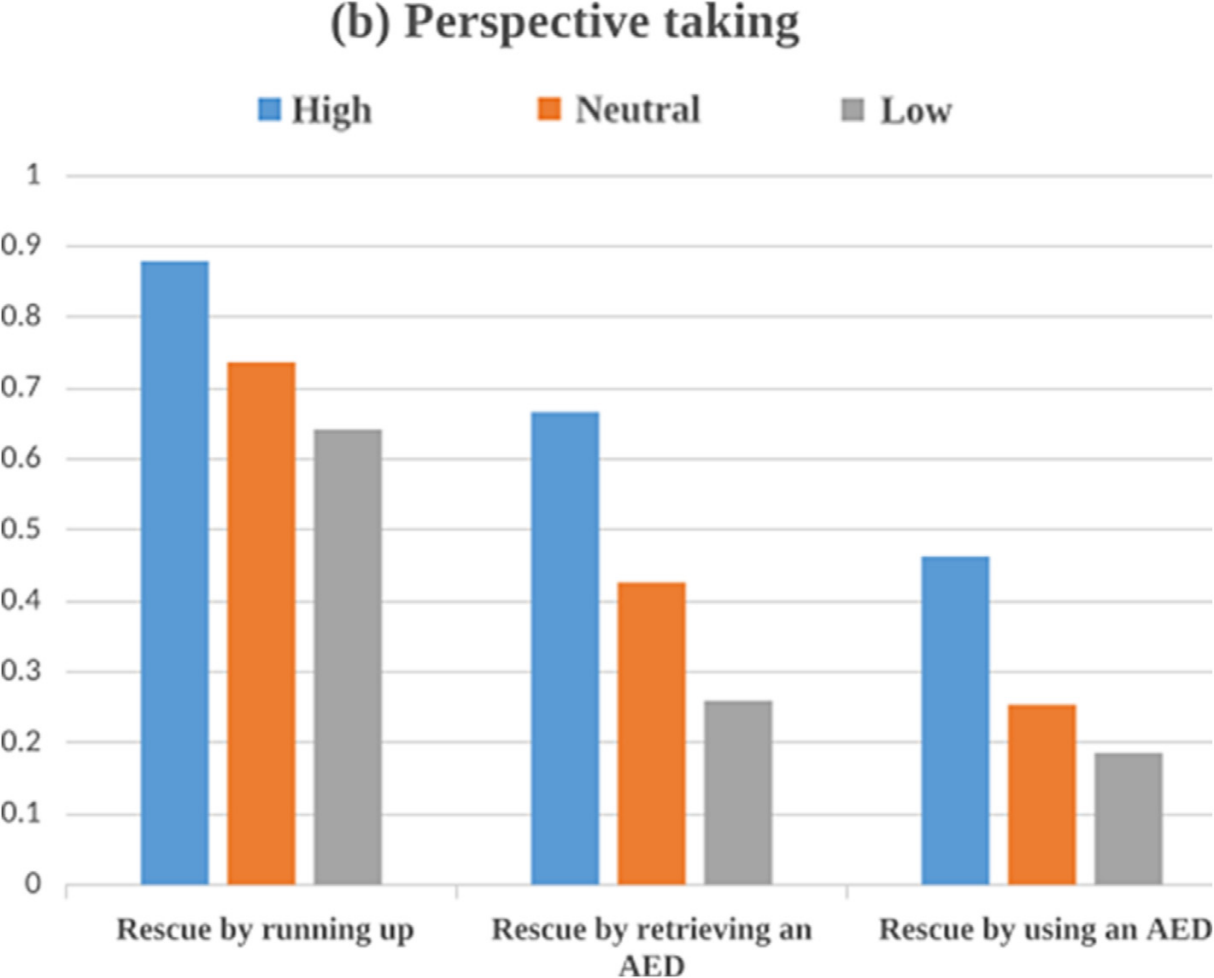

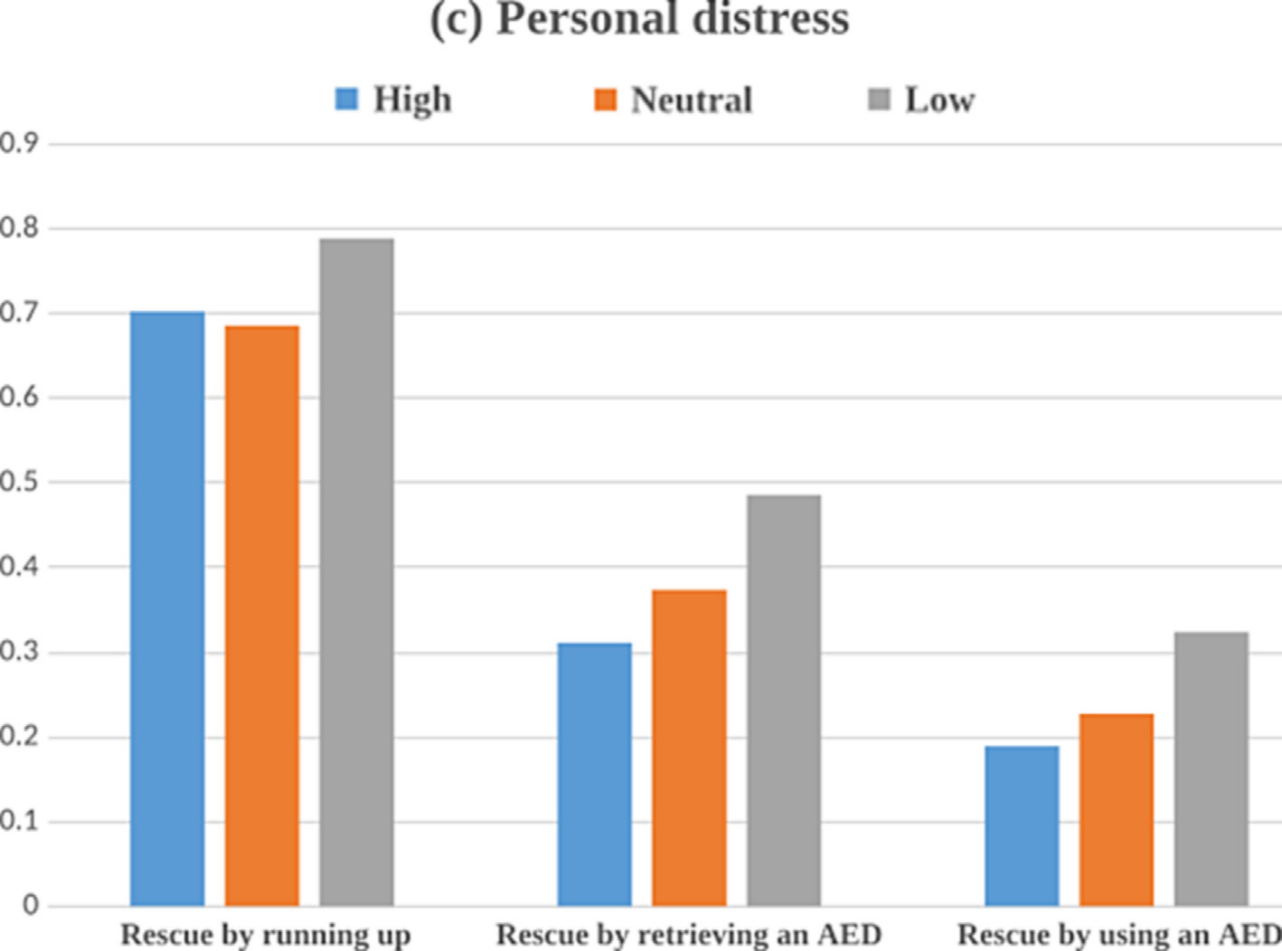
**Willingness to rescue and empathy**.

Fig. 1 further illustrates these patterns across empathy levels. Higher levels of empathic concern and perspective-taking were associated with greater willingness across all three rescue behaviors. For example, the proportion reporting high willingness for running up to the victim and calling for help increased across levels of empathic concern, from 55.4% in the low empathy group to 92.1% in the high empathy group. For willingness to use an AED, these percentages were 13.3% and 41.0%, respectively. A similar gradient was observed for perspective-taking, particularly for AED-related actions. In contrast, higher personal distress was associated with less willingness to retrieve and use an AED. For example, the proportion reporting high willingness to do this declined from 32.4% in participants with low personal distress to 18.9% in those with high personal distress.

To quantify these crude associations, univariable binary logistic regression analyses were performed for each rescue behavior (Table 3). Empathic concern was positively associated with high willingness across all three behaviors (ORs: 3.34 for running up, 2.83 for retrieving an AED, and 2.25 for using an AED; all *p <* 0.001)*.* Perspective-taking was also positively associated with all three behaviors (ORs: 2.43, 3.02, and 2.21; all *p* < 0.001). In contrast, personal distress was inversely associated with all three behaviors (ORs: 0.78, *p* = 0.007; 0.69, *p* < 0.001 and 0.63, *p <* 0.001). Prior AED training was positively associated with all three rescue behaviors, whereas age was associated primarily with willingness to run up.

**Table 3:** Univariable binary logistic regression analyses for high willingness (Likert scale score 4–5 vs 1–3) to perform each rescue behavior.

	**Rescue by running up**		**Rescue by retrieving an AED**		**Rescue by using an AED**	
**Predictor**	**OR (95% CI)**	***P*-value**	**OR (95% CI)**	***P*-value**	**OR (95% CI)**	***P*-value**
Sex	1.02 (0.77–1.35)	0.911	0.73 (0.57–0.95)	0.019	0.65 (0.48–0.88)	0.005
Age	1.17 (1.11–1.23)	<0.001	1.04 (0.99–1.09)	0.083	1.04 (0.99–1.09)	0.170
AED training	1.16 (1.07–1.26)	<0.001	1.27 (1.19–1.37)	<0.001	1.40 (1.29–1.52)	<0.001
Empathic concern	3.34 (2.61–4.27)	<0.001	2.83 (2.27–3.54)	<0.001	2.25 (1.78–2.84)	<0.001
Perspective taking	2.43 (1.90–3.10)	<0.001	3.02 (2.35–3.88)	<0.001	2.21 (1.70–2.87)	<0.001
Personal distress	0.78 (0.65–0.93)	0.007	0.69 (0.59–0.81)	<0.001	0.63 (0.52–0.75)	<0.001

*Abbreviations:* OR, odds ratio; CI, confidence interval; AED, automated external defibrillator.

“Rescue by running up” is an abbreviated label for the first rescue behavior, “running up to the victim or calling for help.”

Each predictor was entered separately in a univariable binary logistic regression model for each rescue behavior.

### 3.4 Multivariable logistic regression analyses

The results of the binary logistic regression analysis are shown in Table 4. Empathic concern and perspective taking were positively associated with willingness to intervene across all three rescue behaviors. In contrast, personal distress showed a distinct pattern: It was inversely associated with willingness to retrieve and use an AED, whereas its association with running up to the victim or calling for help was weaker and not statistically significant.

**Table 4:** Binary logistic regression analyses for high willingness (Likert scale score 4–5 vs 1–3) to perform each rescue behavior.

	**Rescue by running up**		**Rescue by retrieving an AED**		**Rescue by using an AED**	
**Predictor**	**aOR (95% CI)**	***P*-value**	**aOR (95% CI)**	***P*-value**	**aOR (95% CI)**	***P*-value**
Sex	1.28 (0.93–1.77)	0.130	0.69 (0.51–0.93)	0.015	0.60 (0.43–0.85)	0.003
Age	1.36 (1.22–1.53)	<0.001	0.99 (0.89–1.09)	0.828	0.99 (0.88–1.10)	0.819
AED training	1.18 (1.08–1.29)	<0.001	1.27 (1.18–1.38)	<0.001	1.41 (1.29–1.53)	<0.001
Empathic concern	2.42 (1.87–3.14)	<0.001	2.21 (1.74–2.81)	<0.001	2.09 (1.60–2.72)	<0.001
Perspective taking	1.30 (0.99–1.72)	0.058	1.85 (1.43–2.39)	<0.001	1.52 (1.14–2.01)	0.004
Personal distress	0.80 (0.64–1.01)	0.058	0.61 (0.49–0.76)	<0.001	0.64 (0.50–0.82)	<0.001

*Abbreviations:* aOR, adjusted odds ratio; CI, confidence interval; AED, automated external defibrillator.

“Rescue by running up” is an abbreviated label for the first rescue behavior, “running up to the victim or calling for help.”

All models were adjusted for age, sex, AED training experience, and the three empathy dimensions.

Empathic concern was independently associated with increased odds of high willingness across all rescue behaviors: running up or calling for help (aOR 2.42, CI 1.87–3.14, *p* < 0.001), retrieving an AED (aOR 2.21, CI 1.74–2.81, *p* < 0.001), and using an AED (aOR 2.09, CI 1.60–2.72, *p* < 0.001**)**. Perspective-taking was positively associated with willingness to retrieve (aOR 1.85, CI 1.43–2.39, *p* < 0.001) and use an AED (aOR 1.52, CI 1.14–2.01, *p* < 0.003), but not run up (aOR 1.30, CI 0.99–1.72, *p* = 0.058). Personal distress was inversely associated with willingness to retrieve and use an AED (aOR 0.61, CI 0.49–0.76, *p* < 0.001; aOR 0.64, CI 0.50–0.82, *p* < 0.001, respectively) but not with willingness to run up (aOR 0.80, CI 0.64–1.01, *p* = 0.058).

In addition to psychological variables, several demographic factors were associated with willingness to intervene. Female sex was associated with lower willingness to retrieve and use an AED, whereas age was positively associated with running up but not with AED-related actions. Prior AED training was positively associated with willingness to run up to the victim and AED-related actions. Importantly, empathic concern, perspective-taking, and personal distress remained independently associated with willingness to intervene after adjusting for these factors.

## 4 Discussion

This study examined psychological factors associated with bystanders’ willingness to intervene during OHCA, with a particular focus on three dimensions of empathy: empathic concern, perspective-taking, and personal distress. Three key findings emerged:

First, the proportion of participants reporting high willingness was progressively lower for rescue actions requiring greater involvement and responsibility: the highest levels were observed for running up to the victim or calling for help, followed by retrieving an AED, and using an AED. This highlights a potential explanation for why widespread AED availability does not necessarily translate into frequent real-world use, as willingness may decline for actions perceived as more demanding. Consistent with prior literature, bystander intervention has been conceptualized as involving multiple psychological barriers and varying action thresholds rather than a single binary decision.^23^

Second, empathic concern and perspective-taking were positively associated with willingness to intervene. Empathic concern appeared to function as a general motivational factor across all three rescue behaviors, including low-threshold actions such as approaching the victim. In contrast, perspective-taking showed stronger associations with higher-threshold behaviors (AED retrieval and use). This pattern suggests that a cognitive understanding of the victim’s situation becomes increasingly important as actions require greater responsibility and initiative. Psychological theory and empirical studies have suggested that cognitive empathy facilitates complex helping behaviors that require situational appraisal and anticipation of consequences.^24^, ^25^

Third, personal distress demonstrated the opposite pattern. Higher levels of personal distress were associated with reduced willingness to retrieve and use an AED, whereas no significant association was observed for initial engagement behaviors. Personal distress reflects self-focused emotional discomfort when confronted with another person’s suffering. Previous psychological research has shown that such emotional arousal may promote avoidance rather than prosocial action.^26^ Qualitative evidence from lay responders performing CPR further highlights the fact that intense emotional reactions, fear, and uncertainty are common during actual resuscitation attempts.^27^ Our findings extend this literature by suggesting that personal distress may function as a psychological barrier, specifically for high-stakes rescue behaviors such as AED-related actions.

These findings have important implications for public education and training. Traditional AED and BLS programs primarily emphasize technical skills and procedural knowledge.^9^, ^10^, ^11^, ^12^ However, accumulating evidence suggests that psychological readiness is critical to real-world bystander action.^23^, ^28^ Simulation-based training using emotionally realistic scenarios has been proposed to help participants experience and normalize stress responses in a safe environment, thereby improving confidence and reducing hesitation.^28^ In addition, educational approaches that incorporate perspective-taking exercises or victim-centered framing may strengthen cognitive empathy and enhance motivation for higher-threshold behaviors such as AED retrieval and use. Recent literature supports the need to integrate psychological components into resuscitation education. A systematic review of the psychological and behavioral factors influencing CPR initiation highlighted fear, emotional distress, and lack of perceived capability as major barriers to action.^26^ Qualitative synthesis studies have also emphasized the importance of preparing laypersons for the emotional experience of resuscitation, as well as the technical aspects.^27^ Although further empirical evaluation is required, these emerging approaches are conceptually consistent with our findings and suggest that incorporating psychological elements into conventional AED and BLS training may be a promising approach to improve real-world bystander interventions.

This study has some limitations. First, willingness was assessed using a hypothetical scenario, perhaps not fully reflecting actual behavior in real emergencies. Further, although the scenario described a collapsed, unresponsive person with abnormal breathing, intended to represent a suspected cardiac arrest, this was not explicitly stated to participants. The questionnaire also did not specify AED proximity or whether “using an AED” referred specifically to pad application or shock delivery. These ambiguities may have influenced participants’ interpretations of the scenario and should be considered.

Second, an additional limitation concerns the wording of the first rescue behavior item. In the original Japanese questionnaire, this item broadly referred to taking initial action to assist (e.g., running up to the victim or calling for help), rather than a single narrowly defined behavior. As a result, participants may have interpreted this item in different ways, such as approaching the victim directly, verbally calling out to nearby others, or seeking assistance elsewhere. This item may therefore have functioned as a broad or partially double-barreled measure of initial engagement, and the findings for this first rescue behavior should be interpreted with caution. Future studies should use more behaviorally specific and separately measured items to reduce ambiguity. Third, the cross-sectional design precludes causal inferences. Fourth, participants were recruited from an online panel, limiting generalizability. Fifth, although empathy was measured using validated items; cultural norms regarding public intervention and social responsibility in Japan may influence both empathy and willingness to act, which should be considered when interpreting the findings. Nevertheless, a large nationwide sample and a theoretically grounded conceptualization of rescue behaviors with increasing psychological demands strengthen the relevance of the findings.

In conclusion, empathy is a multidimensional psychological factor associated with bystanders’ willingness to intervene during cardiac arrest. While empathic concern and perspective-taking facilitate intervention, personal distress independently inhibits AED-related actions. Addressing these psychological barriers may be essential for improving bystander AED use and ultimately increasing survival after OHCA.

## Data statement

Data will be made available on request to the corresponding author.

## CRediT authorship contribution statement

**Koichi Takaishi:** Writing – review & editing, Writing – original draft, Validation, Supervision, Project administration, Methodology, Investigation, Formal analysis, Data curation, Conceptualization. **Hideko Kono:** Writing – review & editing, Writing – original draft, Validation, Supervision, Project administration, Methodology, Investigation, Funding acquisition, Formal analysis, Data curation, Conceptualization. **Masaya Onuma:** Writing – review & editing, Validation, Supervision, Project administration, Methodology, Investigation, Formal analysis, Data curation, Conceptualization. **Michi Fukushima:** Writing – review & editing, Validation, Supervision, Project administration, Methodology, Investigation, Data curation. **Ryosuke Takeuchi:** Writing – review & editing, Validation, Supervision, Project administration, Methodology, Investigation, Data curation.

## Funding

This work was supported in part by JSPS KAKENHI [grant numbers JP20H01527 and JP24K00281].

## Declaration of competing interest

The authors have no conflicts of interest to declare.

## Appendix A Supplementary material

Supplementary Data 1 Supplementary Table S1. Items of the three IRI subscales used in this study.
