# Supplementary material for: Psychological barriers to bystander AED use: Associations of empathy with willingness to perform bystander rescue behaviors
Source: Resusc Plus. 2026 Jun 23;30:101394. doi: 10.1016/j.resplu.2026.101394 (PMC13351882; doi:10.1016/j.resplu.2026.101394)
Supplement: Supplementary Data 1 — Supplementary Table S1. Items of the three IRI subscales used in this study. [file mmc1.docx]

**Supplementary Material**

Supplementary Table S1. Items of the three IRI subscales used in this study

Items were drawn from the Japanese version of the Interpersonal Reactivity Index (IRI) developed by Himichi et al. [22], based on the subscales used in this study. The English item wording corresponds to the original IRI items.

(Empathic concern)

1. I often have tender, concerned feelings for people less fortunate than me.

2. When I see someone being taken advantage of, I feel kind of protective towards them.

3. I am often quite touched by things that I see happen.

4. I would describe myself as a pretty soft-hearted person.

(Perspective-taking)

1. I try to look at everybody's side of a disagreement before I make a decision.

2. I sometimes try to understand my friends better by imagining how things look from their

perspective.

3. I believe that there are two sides to every question and try to look at them both.

4. When I'm upset at someone, I usually try to "put myself in his shoes" for a while.

5. Before criticizing somebody, I try to imagine how I would feel if I were in their place.

(Personal distress)

1. In emergency situations, I feel apprehensive and ill-at-ease.

2. I sometimes feel helpless when I am in the middle of a very emotional situation.

3. Being in a tense emotional situation scares me.

4. I tend to lose control during emergencies.

5. When I see someone who badly needs help in an emergency, I go to pieces.
